# Prion pathogenesis is unaltered following down-regulation of SIGN-R1

**DOI:** 10.1016/j.virol.2016.08.005

**Published:** 2016-10

**Authors:** Barry M. Bradford, Karen L. Brown, Neil A. Mabbott

**Affiliations:** The Roslin Institute and R(D)SVS, University of Edinburgh, Easter Bush, Midlothian EH25 9RG, UK

**Keywords:** CNS, central nervous system, FDC, follicular dendritic cell, IHC, immunohistochemistry, IV, intravenous, MARCO, macrophage receptor with collagenous structure, MZ, splenic marginal zone, PET., paraffin-embedded tissue, PK, proteinase K, PrP, prion protein, SIGN-R1, specific intercellular adhesion molecule-3-grabbing non-integrin related 1, vCJD, variant Creutzfeldt-Jakob disease, Prions, Transmissible spongiform encephalopathies, Specific intercellular adhesion molecule-3-grabbing non-integrin related 1 (SIGN-R1), Marginal zone, Macrophage, Spleen, Follicular dendritic cell

## Abstract

Prion diseases are infectious neurodegenerative disorders characterised by accumulations of abnormal prion glycoprotein in affected tissues. Following peripheral exposure, many prion strains replicate upon follicular dendritic cells (FDC) in lymphoid tissues before infecting the brain. An intact splenic marginal zone is important for the efficient delivery of prions to FDC. The marginal zone contains a ring of specific intercellular adhesion molecule-3-grabbing non-integrin related 1 (SIGN-R1)-expressing macrophages. This lectin binds dextran and capsular pneumococcal polysaccharides, and also enhances the clearance of apoptotic cells via interactions with complement components. Since prions are acquired as complement-opsonized complexes we determined the role of SIGN-R1 in disease pathogenesis. We show that transient down-regulation of SIGN-R1 prior to intravenous prion exposure had no effect on the early accumulation of prions upon splenic FDC or their subsequent spread to the brain. Thus, SIGN-R1 expression by marginal zone macrophages is not rate-limiting for peripheral prion disease pathogenesis.

## Highlights

• Prions replicate on follicular dendritic cells before infecting the brain.
• The splenic marginal zone aids the delivery of prions to follicular dendritic cells.
• Marginal zone macrophages express SIGN-R1 highly.
• Prion pathogenesis was studies in the transient absence of SIGN-R1 expression.
• SIGN-R1 expression by marginal zone macrophages does not influence prion pathogenesis.

## 1 Introduction

Prion diseases (or transmissible spongiform encephalopathies) are a unique group of subacute, fatal, infectious neurodegenerative disorders. During prion disease, aggregations of PrP^Sc^, an abnormally folded isoform of the host cellular prion glycoprotein (PrP^C^) accumulate in affected tissues (Prusiner, 1982). Prion infectivity co-purifies with PrP^Sc^ and is considered to constitute the major component of the infectious agent (Bolton et al., 1982, Legname et al., 2004, Wang et al., 2010). Once prions reach the central nervous system (CNS) they cause extensive neuropathology which is characterised by the deposition of PrP^Sc^, extensive neurodegeneration and vacuolation (spongiosis), as well as reactive glial responses in both astrocytes and microglia.

Many natural prion diseases including natural sheep scrapie, bovine spongiform encephalopathy in cattle, chronic wasting disease in cervids, and variant Creutzfeldt-Jakob disease (vCJD) in humans, are acquired by peripheral exposure, such as oral consumption of food or pasture contaminated with prions. In humans, accidental iatrogenic prion transmission has also occurred. For example, in the UK four cases of vCJD have been reported in recipients of blood or blood products derived from vCJD-infected donors (Health Protection Agency, 2009; Llewelyn et al., 2004, Peden et al., 2004, Wroe et al., 2006). After peripheral exposure the prions often accumulate and replicate upon the surfaces of PrP^C^-expressing follicular dendritic cells (FDC) within the B-cell follicles of secondary lymphoid tissues (McCulloch et al., 2011). The replication of prions upon FDC is critical for their efficient transmission to the nervous system, termed *neuroinvasion* (Mabbott et al., 2000, Montrasio et al., 2000). Once the prions have been amplified on FDC above the threshold required to achieve neuroinvasion (Mabbott, 2012) they subsequently infect peripheral nerves within the secondary lymphoid tissues, and spread along fibres of both the sympathetic and parasympathetic nervous systems to enter the CNS were they ultimately cause neurodegeneration (Beekes and McBride, 2007, Glatzel et al., 2001, McBride et al., 2001, Prinz et al., 2003).

FDC are large, tissue-fixed, non-motile, stromal-derived cells that reside within the B-follicles (Heesters et al., 2014, Krautler et al., 2012). A thorough understanding of the cellular and molecular mechanisms which facilitate the efficient delivery of prions from the site of exposure to FDC will help identify novel targets for therapeutic or prophylactic intervention. FDC characteristically trap and retain native antigen on their surfaces in the form of immune complexes, consisting of antigen-antibody and/or opsonizing complement components (Fang et al., 1998, Heesters et al., 2014, Taylor et al., 2002). The marginal zone (MZ) surrounding the white pulp regions in the spleen contains a channel of sinus-lining cells through which the blood percolates on its way to the red pulp (Mebius and Kraal, 2005). Attached to this network are specific populations of macrophages and B cells which continually survey the blood-stream for pathogens, antigens and apoptotic cells. These cell populations also regulate the efficient delivery of blood-borne antigens and immune-complexes to the FDC within the splenic B-cell follicles (Arnon et al., 2013, Cinamon et al., 2008, Ferguson et al., 2004, You et al., 2011). Indeed, the delivery of blood-borne antigens to FDC by MZ B cells is impaired in the absence of MZ macrophages (You et al., 2011). An intact MZ is also important for the efficient delivery of prions to FDC in the spleen (Brown et al., 2012, Brown and Mabbott, 2014).

The outer layer of the MZ contains a continuous ring of specific intercellular adhesion molecule-3-grabbing non-integrin related 1 (SIGN-R1/CD209b)-expressing MZ macrophages (Kang et al., 2003). This transmembrane C-type lectin plays an important role in the uptake of dextran (Kang et al., 2003) and capsular pneumococcal polysaccharides (Kang et al., 2004). The prion protein is highly glycosylated, and modifications to the glycosylation status can dramatically alter the ability of certain prion strains to infect splenic FDC (Cancellotti et al., 2010). We have also shown that SIGN-R1-expressing MZ macrophages rapidly acquire fluorescently-labelled prion-specific PrP^Sc^ after IV injection (Bradford et al., 2014). MZ macrophages also mediate the clearance of apoptotic cells through interactions between SIGN-R1 and complement component C1q (Prabagar et al., 2013). Prions are also trapped and retained on the surfaces of FDC (Klein et al., 2001, Mabbott and Bruce, 2004, Mabbott et al., 2001, Michel et al., 2012a, Michel et al., 2013, Zabel et al., 2007) and acquired by mononuclear phagocytes such as conventional dendritic cells in association with complement component C1q (Flores-Lagnarica et al., 2009, Michel et al., 2012b). Since, SIGN-R1 plays an important role in the uptake of blood-borne polysaccharide antigens, and MZ macrophages aid the delivery of certain antigens to FDC in B-cell follicles, a well-characterised *in vivo* method of antibody-mediated SIGN-R1 down-regulation (Gonzalez et al., 2010, Kang et al., 2004, Kang et al., 2003) was used here to test the hypothesis that SIGN-R1-expression in MZ macrophages plays an important role in intravenous (IV) prion disease pathogenesis.

## 2 Materials and methods

### 2.1 Mice and transient SIGN-R1 down-regulation

Age and sex-matched C57BL/6J mice (Charles River Laboratories, Harlow, UK) were used throughout this study and maintained under specific pathogen-free conditions. To transiently down-regulate SIGN-R1 expression *in vivo*, mice were injected IV with 100 µg of hamster anti-mouse SIGN-R1 monoclonal antibody (mAb; clone 22D1, eBioscience, Hatfield, UK) as described (Gonzalez et al., 2010, Kang et al., 2006, Kang et al., 2004). A parallel group of mice were treated with 100 µg of purified, isotype-matched, non-specific hamster IgG (eBioscience) as a control (termed control-Ig, hereinafter). All studies and regulatory licences were approved by the University of Edinburgh's ethics committee and carried out under the authority of a UK Home Office Project License.

### 2.2 Prion exposure and disease monitoring

Twenty four hours after antibody treatment, mice were infected with a limiting dose of ME7 scrapie prions by IV injection with 20 µl of a 0.1% (weight/volume) brain homogenate prepared from mice with terminal prion disease. Some mice were culled 35 d after prion exposure and spleens taken for further analysis. The remaining mice were observed for signs of clinical prion disease and culled at a standard clinical end-point as described (Brown et al., 2012). Clinical prion disease diagnosis was confirmed by the histopathological assessment of vacuolation (spongiform pathology) in the brain (Fraser and Dickinson, 1968). For the construction of lesion profiles, haematoxylin and eosin (H&E)-stained brain sections were scored for the presence and severity (scale 0–5) of prion-disease-specific vacuolation in nine grey matter brain areas: G1, dorsal medulla; G2, cerebellar cortex; G3, superior colliculus; G4, hypothalamus; G5, medial thalamus; G6, hippocampus; G7, septum; G8, cerebral cortex; G9, forebrain cerebral cortex.

### 2.3 Immunohistochemical (IHC) analysis

For the detection of prion disease-specific PrP (PrP^d^) in brains and spleens, tissues were first fixed in periodate–lysine–paraformaldehyde fixative and embedded in paraffin wax. Sections (6 µm in thickness) were deparaffinized, and pre-treated to enhance the detection of PrP^d^ by hydrated autoclaving (15 min, 121 °C), and subsequently immersion in 98% formic acid (McBride et al., 1992). Spleen sections were then immunostained with 1B3 PrP-specific polyclonal antiserum (Farquhar et al., 1989), and brain sections were immunostained with mouse anti-PrP-specific mAb (clone 6H4; Prionics, Schlieren-Zurich, Switzerland). Paraffin-embedded tissue (PET) immunoblot analysis was used to confirm that the PrP^d^ detected by IHC was prion disease-specific, proteinase K (PK)-resistant PrP^Sc^ (Schulz-Schaeffer et al., 2000). For the detection of FDC in periodate–lysine–paraformaldehyde-fixed spleens, deparaffinized sections were first pretreated with Target Retrieval Solution (Dako, Glostrup, Denmark) and subsequently immunostained with rat anti-mouse CD21/35 (clone 7G6; BD Biosciences, Oxford, UK). For the detection of microglia, deparaffinised brain sections were immunostained with rabbit anti-allograft inflammatory factor 1 (AIF1/Iba1; Wako Chemicals GmbH, Neuss, Germany), and astrocytes were detected using rabbit anti-glial fibrillary acidic protein (GFAP; Dako). To detect FDC and macrophages, spleens were flash frozen at the temperature of liquid nitrogen and sections (10 µm in thickness) were cut on cryostat and immunostained with the following antibodies: FDC were detected using rat anti-mouse CD35 mAb (clone 8C12; BD Biosciences); marginal metallophilic macrophages were detected using rat anti-mouse sialoadhesin/CD169 mAb (clone MOMA-1; Bio-Rad AbD Serotec, Oxford, UK); MZ macrophages were detected using hamster anti-mouse SIGN-R1 mAb (clone 22D1), rat anti-mouse SIGN-R1 mAb (clone ER-TR9; Bio-Rad AbD Serotec) or rat anti-mouse MARCO (clone ED31; Bio-Rad AbD Serotec).

For light microscopy, biotin-conjugated species-specific secondary antibodies (Stratech, Soham, UK) were subsequently applied, and immunolabelling was revealed using horseradish peroxidase-conjugated to the avidin-biotin complex (Vector Laboratories, Peterborough, UK) and visualised with 3,3′-diaminobenzidine (DAB; Sigma, Poole, UK). Sections were counterstained with haematoxylin to detect cell nuclei. For fluorescence microscopy, species-specific secondary antibodies coupled to Alexa-Fluor 488, Alexa-Fluor 594 or Alexa-Fluor 647 dyes were used (Invitrogen, Paisley, UK). Sections were mounted in fluorescent mounting medium (Dako) and examined using a Zeiss LSM5 or LSM710 confocal microscopes (Zeiss, Welwyn Garden City, UK). Image analyses were performed using Zen (Zeiss) or ImageJ software (http://imagej/nih.gov/ij) on a minimum of six animals per group and six observations per animal, for 72 individual images analysed per comparison.

### 2.4 *In vivo* assessment of antigen trapping

Twenty four hours after antibody treatment mice were passively immunized by IV injection with either 100 µl of pre-formed peroxidase–anti peroxidase (PAP) immune complexes (Sigma) (Brown et al., 2012, McCulloch et al., 2011) or fluorescein isothiocyanate (FITC)-labelled 70 kDa dextran (Sigma) (Kang et al., 2003). Spleens were collected 24 h after injection. PAP immune complexes were visualised by IHC using AlexaFluor 488-conjugated goat anti-rabbit IgG. The magnitude of the FDC-associated PAP immune complexes in spleens from each group was then determined using ImageJ software as described (Brown et al., 2012). Spleens from 6 mice from each group were analysed. Typically from each spleen, 4 sections were studied, and on each section, data from 3 randomly chosen 1000- by 1000-µm fields of view were collected.

### 2.5 Immunoblot detection of PrP^Sc^

Brain homogenates (10% weight/volume) were prepared in NP40 lysis buffer (1% NP40, 0.5% sodium deoxycholate, 150 mM NaCl, 50 mM TrisHCL [pH 7.5]) and incubated at 37 °C for 1 h with 20 µg/ml PK. Digestions were halted by addition of 1 mM phenylmethylsulfonyl fluoride. Samples were then subjected to electrophoresis through 12% Tris-glycine polyacrylamide gels (Nupage, Life Technologies) and transferred to PVDF membranes by semi-dry blotting. PrP was detected using anti-mouse PrP-specific mAb 7A12 (Yin et al., 2007) followed by horseradish peroxidase-conjugated goat anti-mouse antibody (Jackson Immunoresearch) and visualised chemiluminescence (BM Chemiluminescent substrate kit, Roche, Burgess Hill, UK).

### 2.6 Statistical analysis

Statistical analyses were performed using Minitab 16 software (Minitab Ltd., Coventry, UK). Survival times after prion exposure and immunofluorescence analysis quantification data were tested for equal variances and analysed by two-sample *t*-test. Vacuolation profile data were analysed via analysis of variance and grouped via Tukey's post hoc testing. Data are presented as mean±SEM. *P*<0.05 was accepted as significant.

## 3 Results

### 3.1 Transient down-regulation of SIGN-R1 on MZ macrophages

**Fig. 1:**
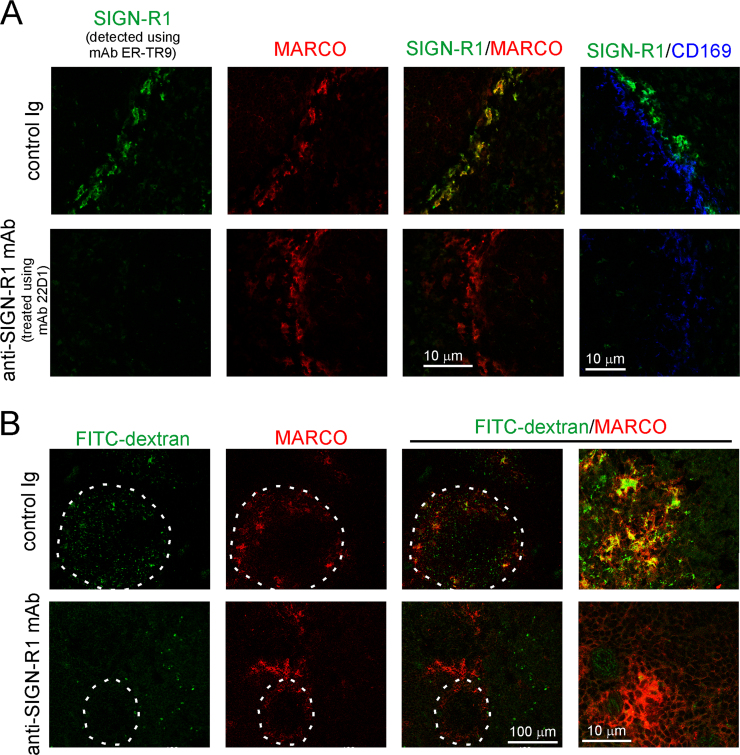
Transient down-regulation of SIGN-R1 on MZ macrophages. Panel A. Mice were injected IV with anti-SIGN-R1-specific mAb 22D1 or control Ig and spleens collected 24 h later. In spleens from control Ig-treated mice (upper row) MZ macrophages expressing SIGN-R1 (green, detected with anti- SIGN-R1 mAb clone ER-TR9) and MARCO (red) were readily detected in the MZ. SIGN-R1 expression on MZ macrophages was down-regulated in the spleens of anti-SIGN-R1 mAb-treated mice (lower row). Anti-SIGN-R1 mAb treatment did not affect the expression of CD169 on the marginal metallophilic macrophages (blue, right-hand panels). Panel B. Twenty four hours after antibody treatment mice were injected IV with FITC-70 kDa dextran (FITC-dextran, green) and spleens collected 24 h later. MZ macrophages (MARCO^+^ cells, red) in the spleens of anti-SIGN-R1 mAb-treated mice were unable to retain FITC-dextran (lower row). Dotted lines indicate the boundary of the MZ. *n*=6 mice/group.

To determine the contribution of SIGN-R1 in prion disease pathogenesis, the expression of this receptor on MZ macrophages was transiently down-regulated prior to IV prion exposure (Gonzalez et al., 2010, Kang et al., 2006, Kang et al., 2004). Mice (*n*=6/group) were injected IV with anti-SIGN-R1-specific mAb 22D1 and spleens collected 24 h later. A parallel group of mice received an isotype matched, non-specific hamster IgG as a control (control Ig). To avoid the possibility that treatment with anti-SIGN-R1-specific mAb 22D1 might mask epitopes on SIGN-R1 in the spleens of treated mice, an alternative SIGN-R1-specific mAb (rat anti-mouse SIGN-R1 mAb clone ER-TR9) was used for IHC analysis. In the spleens of control Ig-treated mice SIGN-R1-expressing MZ macrophages were readily detected in the outer layer of the MZ (Fig. 1A). IHC analysis also confirmed that these MZ macrophages co-expressed high levels of macrophage receptor with collagenous structure (MARCO; Fig. 1A, upper right-hand panel). In contrast, the expression of SIGN-R1 on MZ macrophages was dramatically down-regulated in the spleens of anti-SIGN-R1 mAb-treated mice (Fig. 1A, lower row). Indeed, consistent with previous reports (Gonzalez et al., 2010, Kang et al., 2006, Kang et al., 2004) SIGN-R1 expression was transiently undetectable in the spleen after anti-SIGN-R1 mAb treatment. This effect was not due to depletion of the MZ macrophages, as the expression of MARCO on these cells was unaffected (Fig. 1A). Anti-SIGN-R1 mAb treatment did not affect the expression of CD169 (sialoadhesin) on the marginal metallophilic macrophages in the inner layer of the MZ (Fig. 1A, lower right-hand panels), confirming that the effects of treatment were specific to the MZ macrophages.

The uptake of the dextran polysaccharides by MZ macrophages is mediated by SIGN-R1 (Kang et al., 2006, Kang et al., 2004). As anticipated the MZ macrophages in the spleens of control Ig-treated mice were able to trap 70 kDa FITC-dextran after IV injection (Fig. 1B, upper row). In contrast, the MARCO-expressing MZ macrophages in the spleens of anti-SIGN-R1 mAb-treated mice were unable to acquire and trap dextran (Fig. 1B, lower row). These data confirm that anti-SIGN-R1 mAb treatment transiently down-regulates SIGN-R1 expression in MZ macrophages. Previous studies show that the anti-SIGN-R1 mAb treatment as used here causes a selective, prolonged but transient down-regulation of SIGN-R1 expression on MZ macrophage for approximately 5–15 days (Kang et al., 2004).

### 3.2 Effect of SIGN-R1 down-regulation on FDC

**Fig. 2:**
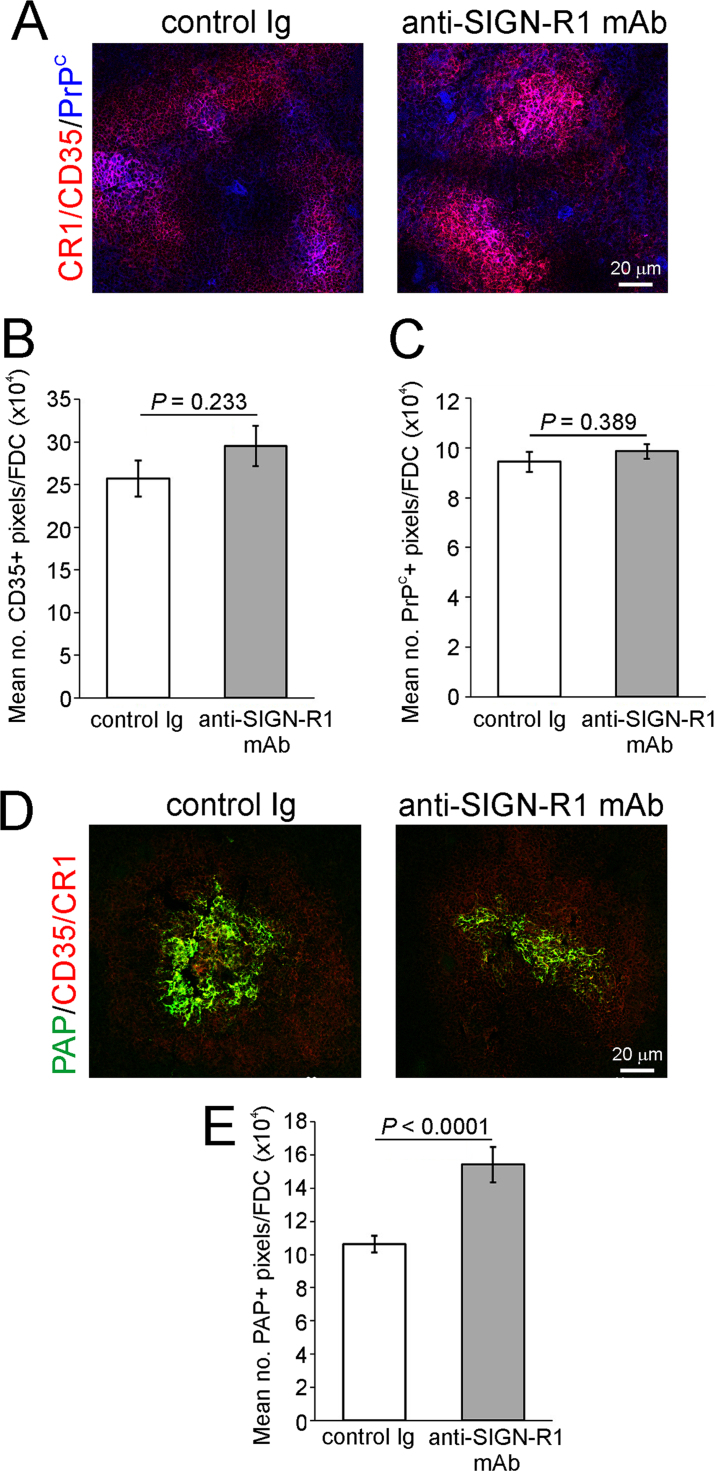
Transient down-regulation of SIGN-R1 on MZ macrophages does not adversely affect FDC status. Panel A. IHC analysis suggested there was no observable difference in the expression of CR1/CD35 (red) or cellular PrP^C^ (blue) in FDC in spleens from anti-SIGN-R1 mAb- or control Ig-treated mice. Panels B and C. Morphometric analysis confirmed that the magnitude of the CR1/CD35- and PrP^C^-specific immunostaining observed in the spleens from the anti-SIGN-R1 mAb or control Ig mice was similar. Panel D. Mice were injected IV with anti-SIGN-R1-specific mAb 22D1 or control Ig, and 24 h later passively immunized with preformed PAP immune complexes (*n*=6 mice/group). Twenty four hours after treatment the presence of immune complexes (PAP, green) upon FDC (CR1/CD25^+^ cells, red) was determined by IHC. Panel E. Morphometric analysis suggested the amount of PAP trapped on the surfaces of the FDC in the spleens of anti-SIGN-R1 mAb-treated mice was significantly greater when compared to control Ig-treated mice (*P*<0.0001).

Since the replication of prions upon PrP^C^-expressing FDC is obligatory for their efficient neuroinvasion (Mabbott et al., 2000, Montrasio et al., 2000), we determined the effect of SIGN-R1 down-regulation on FDC status. FDC characteristically express high levels of complement receptor 1 (CR1/CD35) and PrP^C^ (McCulloch et al., 2011, Zabel et al., 2007). IHC and morphometric analysis suggested there was no observable difference in the size of the FDC networks in spleens from control-Ig and anti-SIGN-R1 mAb treated mice (Fig. 2A and B). The expression of PrP^C^ upon the FDC was also similar in spleens from mice from each treatment group (Fig. 2A and C).

To determine whether SIGN-R1 down-regulation affected immune complex retention on FDC, control Ig- and anti-SIGN-R1-treated mice (*n*=6/group) were passively immunized by IV injection with preformed PAP immune complexes, and the presence of FDC-associated immune complexes analysed by IHC 24 h later. High levels of PAP-containing immune complexes were detected in association with CD35-expressing FDC in the spleens of mice from each group (Fig. 2D). However, morphometric analysis suggested a small but significant increase in the magnitude of immune complex trapping on FDC in the spleens of anti-SIGN-R1-treated mice (*P*<0.0001, two-sample *t*-test, *n*=36 FDC/group; Fig. 2E). Together, these data show that transient SIGN-R1 down-regulation does not adversely affect FDC status and function.

### 3.3 Effect of SIGN-R1 down-regulation on prion accumulation in the spleen

**Fig. 3:**
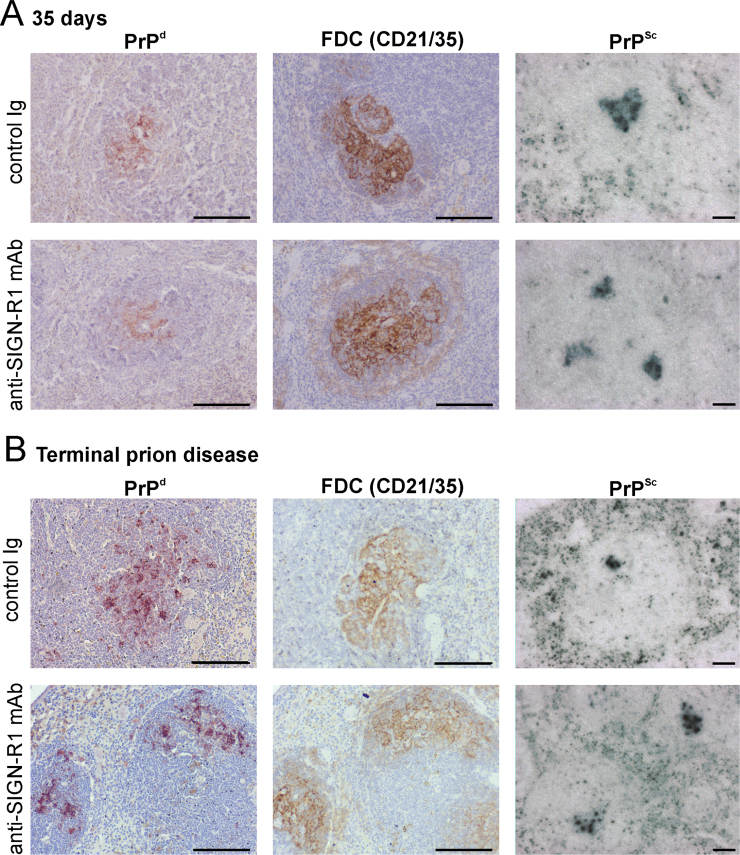
Transient down-regulation of SIGN-R1 on MZ macrophages on does not impair the accumulation of PrP^Sc^ upon FDC in the spleen. Mice were injected IV with anti-SIGN-R1- mAb or control Ig and 24 h later injected IV with ME7 scrapie prions. Spleens were collected 35 days after IV prion injection (Panel A) or at the terminal stage of disease (Panel B). At each time point abundant prion disease-specific PrP (PrP^d^, brown, left-hand column) accumulated in association with FDC (CD21/35^+^ cells, brown, middle column) in the spleens of mice from each treatment group. Analysis of adjacent sections by PET immunoblot analysis confirmed the presence of prion-disease specific, relatively proteinase K-resistant PrP^Sc^ (black, right-hand column).

Within 35 days of IV prion exposure high levels of prion-specific PrP^Sc^ accumulate upon splenic FDC and are maintained for the duration of the infection (Brown et al., 2012). Here, mice were injected IV with ME7 scrapie prions 24 h after treatment with either control Ig- or anti-SIGN-R1 mAb. The mice were injected with a limiting dose of prions (20 µl of a 0.1% scrapie brain homogenate) as in our previous study (Brown et al., 2012), to avoid the possibility that dose of prions administered was sufficiently high enough to by-pass the requirement for replication within the spleen prior to neuroinvasion. Spleens from 4 mice from each group were collected 35 days later and the effects of SIGN-R1 down-regulation on the early accumulation of PrP^Sc^ on FDC determined. As anticipated, PrP^d^ accumulation upon FDC was readily detected in spleens from control Ig-treated mice (Fig. 3A). PET immunoblot analysis confirmed the PrP^d^ detected by IHC was prion disease-specific, relatively PK-resistant, PrP^Sc^ (Fig. 3A). In spleens from anti-SIGN-R1 mAb-treated mice similar levels of FDC-associated PrP^Sc^ were also detected (Fig. 3A). Furthermore, at the terminal stage of disease, high levels of PrP^Sc^ were maintained upon FDC in the spleens of mice from each treatment group (Fig. 3B). These data show that SIGN-R1 down-regulation did not impede the accumulation of PrP^Sc^ upon FDC in the spleen.

### 3.4 Effect of SIGN-R1 downregulation on prion disease susceptibility

**Fig. 4:**
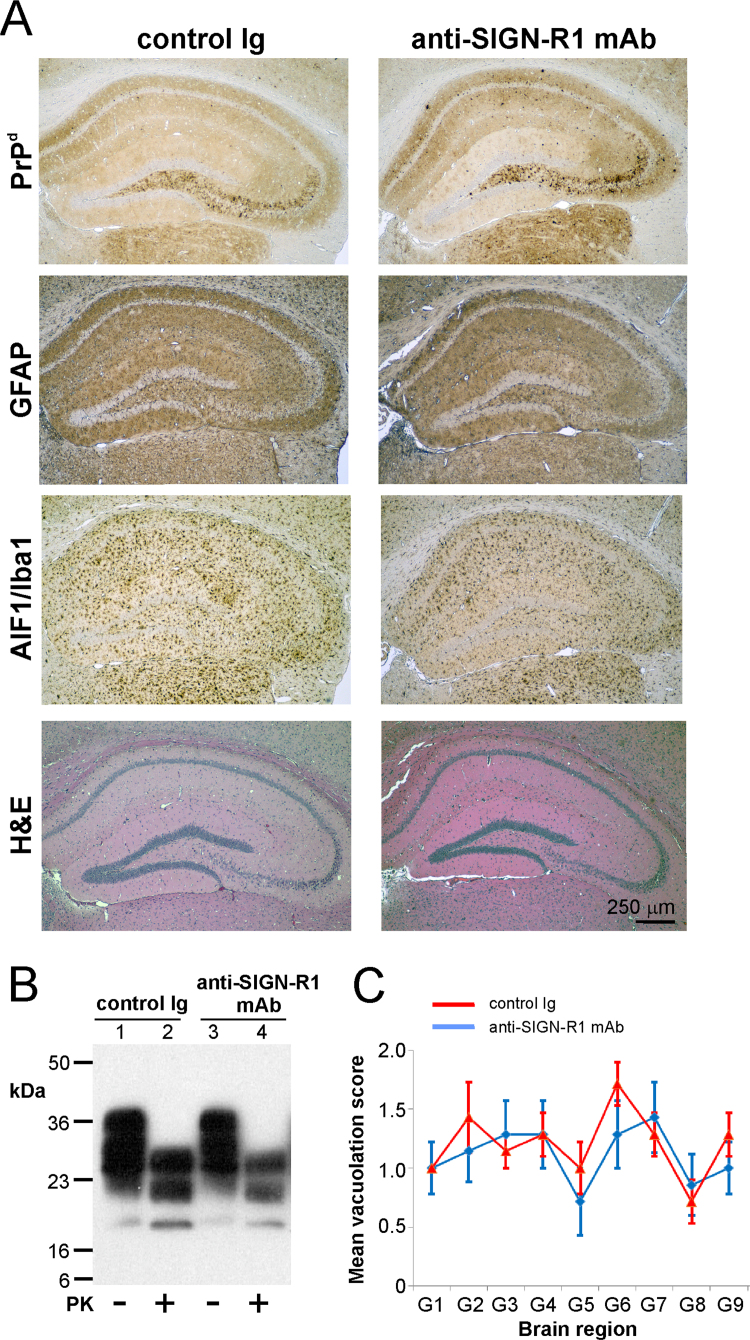
Transient down-regulation of SIGN-R1 on MZ macrophages at the time of IV prion infection does affect the development of neuropathology in the brain at the terminal stage of disease. Mice were injected IV with anti-SIGN-R1 mAb or control Ig and 24 h later injected IV with ME7 scrapie prions (*n*=8 mice/group). Brains were collected at the terminal stage of disease. Panel A. Histopathological analysis showed large accumulations of prion disease-specific PrP^d^ (brown, upper row), reactive astrocytes expressing GFAP (brown, second row), active microglia expressing AIF1/Iba1 (brown, third row) and spongiform pathology (H&E, bottom row) in brains of all terminally-affected control Ig-treated (left-hand column) and anti-SIGN-R1 mAb-treated (right-hand column) mice. Sections were counterstained with haematoxylin to detect cell nuclei (blue). Panel B. Immunoblot analysis of brain tissue homogenates confirmed the presence of high levels of prion-specific, relatively proteinase K (PK)-resistant PrP^Sc^ within the brains of mice from each treatment group. Samples were treated in the presence (+) or absence (−) of PK before electrophoresis. After PK treatment, a typical three-band pattern was observed between molecular mass values of 20–30 kDa, representing unglycosylated, monoglycosylated, and diglycosylated isomers of PrP (in order of increasing molecular mass). Panel C. The severity and distribution of the spongiform pathology (vacuolation) within the brains of all terminally-affected mice from each treatment group was similar. The severity of the vacuolation in each brain was scored on a scale of 1–5 in the following grey matter regions: G1, dorsal medulla; G2, cerebellar cortex; G3, superior colliculus; G4, hypothalamus; G5, thalamus; G6, hippocampus; G7, septum; G8, retrosplenial and adjacent motor cortex; G9, cingulate and adjacent motor cortex.

We next determined the effect of SIGN-R1 down-regulation on disease duration after IV prion exposure. Control Ig- or anti-SIGN-R1-treated mice were injected IV with ME7 scrapie prions (*n*=8/group) and monitored for the clinical signs of prion disease. Regardless of antibody treatment, all mice in each treatment group succumbed to clinical prion disease with similar survival times: control Ig-treated mice 262±7 d; anti-SIGN-R1 treated mice 261±7 d (*P*=0.903, two sample *t*-test). Histopathological analysis confirmed that the brains from all the clinically-affected control Ig- or anti-SIGN-R1 mAb-treated mice displayed the characteristic PrP^d^ accumulation, astrogliosis and microgliosis associated with terminal infection with ME7 scrapie prions (Fig. 4A). Immunoblot analysis confirmed that similar levels of prion disease-specific PrP^Sc^ were present in the brains of the clinically-affected mice from each treatment group (Fig. 4B). Furthermore, the distribution and severity of the spongiform pathology (vacuolation) was also similar in the brains of the clinically-affected, control Ig- or anti-SIGN-R1 mAb-treated mice (Fig. 4C). These data clearly show that SIGN-R1 down-regulation did not significantly influence survival time, disease susceptibility or the development of neuropathology after IV prion exposure.

## 4 Discussion

An intact splenic MZ is important for the efficient delivery of certain antigens and prions to FDC (Brown et al., 2012, Brown and Mabbott, 2014, Cinamon et al., 2008, You et al., 2011). The MZ macrophages within the outer layer of the MZ specifically express high levels of the lectin SIGN-R1. This lectin mediates the uptake of dextran (Kang et al., 2003) and bacterial capsular polysaccharides (Kang et al., 2004), and also aids the clearance of apoptotic cells through interactions with complement component C1q (Prabagar et al., 2013). Since a diverse range of pathogens including HIV (Geijtenbeek and van Kooyk, 2003), ebola virus (Simmons et al., 2003), *Mycobacterium tuberculosis* (Geijtenbeek et al., 2003), and *Leishmania* amastigotes (Colmenares et al., 2002) appear to exploit DC-SIGN (the human homologue of SIGN-R1) to infect host mononuclear phagocytes and/or suppress immune responses, we hypothesised that SIGN-R1 might also mediate the uptake of prions by MZ macrophages. When macrophages are depleted prior to prion exposure, the accumulation of prions in the spleen is enhanced (Beringue et al., 2000). In the absence of SIGN-R1 expression it is plausible that prion accumulation in the spleen and subsequent neuroinvasion would be enhanced, due to decreased sequestration of prions from the blood by MZ macrophages. Certain antigens which have been trapped by MZ macrophages are subsequently acquired by MZ B cells which deliver them the FDC-containing B-cell follicles by MZ B cells (Cinamon et al., 2008, You et al., 2011). This activity is reduced in the absence of MZ macrophages (You et al., 2011). Thus, alternatively, prion accumulation in the spleen and subsequent neuroinvasion might be impaired, due to the less efficient shuttling of prions to FDC.

Here, SIGN-R1-expression was down-regulated *in vivo* on MZ macrophages using a well-characterised mAb-mediated method (Gonzalez et al., 2010, Kang et al., 2004, Kang et al., 2003), and the influence this had on IV prion disease pathogenesis determined. As anticipated, the MZ macrophages in these mice were unable to capture blood-borne dextran particles. However, transient down-regulation of SIGN-R1 had no effect on the early accumulation of prions upon splenic FDC or the subsequent spread of disease to the CNS. Thus, SIGN-R1 expression by MZ macrophages is not rate-limiting for peripheral prion disease pathogenesis. Our data do not exclude a role for MZ macrophages in the initial uptake and processing of prions in the spleen, only that SIGN-R1 expression in these cells is dispensable for this activity. The majority of the cell populations within the mammalian immune system, including mononuclear phagocytes, express cellular PrP^C^ (Mabbott and Bradford, 2015). However, the replication of prions upon splenic FDC is unaffected in mice in which PrP^C^ is expressed only in FDC (McCulloch et al., 2011), indicating that PrP^C^ is itself unlikely to be a major uptake receptor for prions on MZ macrophages.

Although the duration of the SIGN-R1 down-regulation on MZ macrophages was transient, we consider it unlikely that a more prolonged deficiency in SIGN-R1 would significantly influence the early stages of prion disease pathogenesis in the spleen. Immune complexes and complement-opsonized antigens are acquired by MZ macrophages and delivered to FDC within hours of IV injection (Fig. 2) (Cinamon et al., 2008, Kang et al., 2004, Kang et al., 2003). We have also shown that fluorescently-labelled PrP^Sc^ is rapidly acquired by SIGN-R1-expressing MZ macrophages *in vivo* within 1h of IV injection, with smaller amounts already detectable on FDC in the B cell follicles (Bradford et al., 2014). Furthermore, the transient depletion of CD11c^+^ mononuclear phagocytes for approximately 2–4 days dramatically reduces peripheral prion disease susceptibility (Cordier-Dirikoc and Chabry, 2008, Raymond et al., 2007). Previous studies show that anti-SIGN-R1 mAb-treatment causes a selective, prolonged, but transient down-regulation of SIGN-R1 expression on MZ macrophages *in vivo* for approximately 5–15 days (Kang et al., 2004). Together, these observations imply that the duration of the SIGN-R1 down-regulation was more than sufficient to study the potential role of SIGN-R1 expression on MZ macrophages in the early accumulation of prions in the spleen.

MZ B cells can acquire certain antigens from MZ macrophages and mediate their delivery to the FDC (Cinamon et al., 2008, Kang et al., 2003, You et al., 2011). Our data revealed a small but significant increase in the magnitude of PAP immune complexes trapped on the surfaces of FDC in the spleens of anti-SIGN-R1 mAb-treated mice. This is implied that in the transient absence of SIGN-R1 expression, fewer immune complexes were sequestered by MZ macrophages enabling a greater amount to be shuttled to FDC.

In summary, although SIGN-R1 plays an important role in the uptake of certain polysaccharides, complement-opsonized apoptotic cells and a diverse range of microbial pathogens, our data clearly show that SIGN-R1 expression in MZ macrophages is dispensable for the efficient delivery of prions to FDC in the spleen and the subsequent spread of infection to the CNS. A thorough understanding of the cellular and molecular factors within the MZ which regulate the transfer of prions to FDC will aid identification of novel targets for prophylactic intervention in these currently untreatable, devastating, neurodegenerative diseases.
